# Baseline study of the morphological and genetic characteristics of *Haemoproteus* parasites in wild pigeons (*Columba livia*) from paddy fields in Thailand

**DOI:** 10.1016/j.ijppaw.2023.04.003

**Published:** 2023-04-11

**Authors:** Phirom Prompiram, Chalisa Mongkolphan, Kanaporn Poltep, Supatra Chunchob, Narin Sontigun, Theeraphap Chareonviriyaphap

**Affiliations:** a The Monitoring and Surveillance Center for Zoonotic Diseases in Wildlife and Exotic Animals, Faculty of Veterinary Science, Mahidol University, Salaya, Nakhon Pathom, 73170, Thailand; b Mahidol University, Division of Conservation Biology, School of Interdisciplinary Studies, Sai Yok, Kanchanaburi, 71150, Thailand; c Akkhraratchakumari Veterinary College, Walailak University, Nakhon Si Thammarat, 80161, Thailand; d Department of Entomology, Faculty of Agriculture, Kasetsart University, Bangkok, 10900, Thailand

**Keywords:** Cytochrome *b* gene, Genetic diversity, *Haemoproteus columbae*, Molecular character, Morphological character, Wild pigeon

## Abstract

*Haemoproteus columbae* is a common haemosporidian parasite of wild pigeons (*Columba livia*) reported worldwide. In Thailand, the wild pigeon population is increasing due to paddy field monoculture. However, there are limited reports on the presence of *H. columbae* in these pigeon populations. The aim of the study was to characterize *H. columbae* in wild pigeons. A total of 87 wild pigeons were examined using microscopic and molecular methods. *Haemoproteus columbae* was detected in approximately 27.6% of pigeons and their morphological characteristics were described. The partial cytochrome *b* (*cyt b*) gene sequence of *H. columbae* was then characterized into three common lineages (HAECOL1, COLIV03, and COQUI05). By highlighting the morphologic and genetic characteristics of *H. columbae* commonly found in this population of pigeons, this study provides essential regional knowledge about haemosporidian parasites that could benefit future taxonomic and phylogeographic studies.

## Highlights

• We report the morphology and genetics of the *Haemoproteus* parasite found in wild pigeons in paddy fields in Thailand.
• Of 87 wild pigeons, 27.6% had haemosporidian infection.
• Based on morphologic characteristics, the *Haemoproteus* parasite was identified as *Haemoproteus columbae.*
• Genetic analysis revealed three common *cytb* haplotypes: HAECOL1, COLIV03, and COQUI05.

## Graphical abstract

**Figure:**
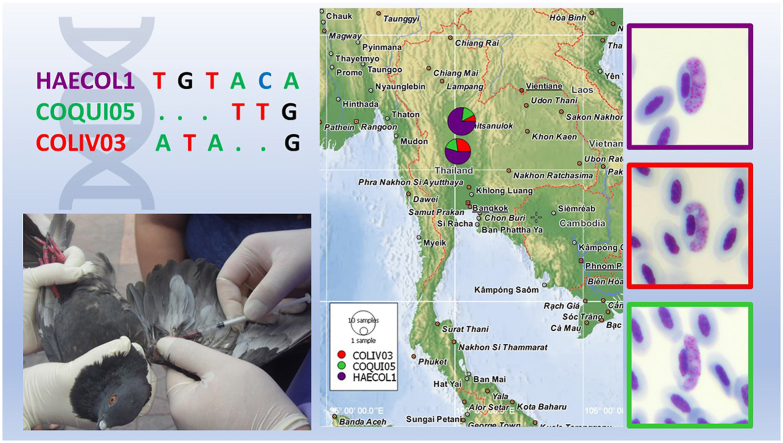

## 1 Introduction

*Haemoproteus columbae* is a haemosporidian parasite found in wild pigeons (*Columba livia*) (Valkiunas et al., 2010). Pigeons infected with *H. columbae* exhibit gametocyte stages: exo-erythrocytic (tissue) meronts, which are found in liver, spleen, and other organs (Earle et al., 1993; Ferrell et al., 2007), can have severe pathological effects (Valkiunas and Iezhova, 2017). The louse fly *Microlynchia pusilla*, *Pseudolynchia canariensis,* and *P. brunnea* are vectors of *H. columbae* (Cepeda et al., 2019; Klei and DeGiusti, 1975; Valkiunas, 2004). Because *H. columbae* together with wild pigeons (*Columba livia*) have been introduced worldwide, they have a wide geographic distribution (Boano et al., 2018; Chagas et al., 2016; Fecchio et al., 2017; Gil-Vargas and Sedano-Cruz, 2019; Nebel et al., 2020; Tang et al., 2018; Yumoto et al., 2021).

In Thailand, wild pigeons are an invasive species (Round, 2019) distributed throughout the country — especially in lowland regions with large areas of paddy fields, such as Nakhon Sawan and Phitsanulok provinces. Wild pigeons are granivorous and frequently invade Thailand's paddy fields, which are increasing to meet rising rice export demands (Batool et al., 2020; Thai Rice Exporters Association, 2000). As a result, the wild pigeon population in Thailand is rapidly growing and widely dispersed in paddy fields (Tang et al., 2018). Wild pigeons are known to harbor various zoonotic pathogens, such as *Chlamydia* bacteria, *Cryptosporidium* protozoa, and *Alphainfluenzavirus influenzae* (Koompapong et al., 2014; Prompiram et al., 2022; Sariya et al., 2015). However, the common epizootic pathogens, such as *H. columbae,* in wild pigeons in Thailand have rarely been studied, with only an unidentified *Haemoproteus* parasite reported in urban areas of the Chiang Mai province (Buranapim et al., 2019). Therefore, it is necessary to better understand Haemosporidia found in the wild pigeon population in Thailand.

This study aimed to identify *H. columbae* species using traditional morphological and molecular methods and to delineate haplotype variation. These findings can serve as a foundation for understanding its distribution in wild pigeons in paddy fields in Thailand. The results will also be useful for defining the molecular characteristics of *H. columbae* in tropical regions – particularly agricultural areas.

## 2 Materials and methods

### 2.1 Study site

This study involved sampling of two sites: 1) the Nakhon Sawan province (15° 34ʹ N, 100° 13ʹ E), which is a lowland area of the Chao Phraya River in upper central Thailand (altitude; 33 m), and 2) the Phitsanulok province (16° 48′ N, 100° 15′ E), which is a flatland area of the Nan Basin with some hills and woods located in lower northern Thailand (altitude: 51 m). Both study sites have a tropical climate with temperatures ranging 23.1 °C–33.2 °C and 26.3 °C–35.8 °C, respectively, as well as a monsoon with average annual precipitation of 1182 and 1358.5 mm, respectively. These sites are appropriate for agriculture like paddy fields that provide food for wild pigeons.

### 2.2 Sample collection

Wild pigeons were captured using mist nets over two trapping days per site during the dry season. Traps were set in mid-December 2018 at the Nakhon Sawan site, and beginning of March 2019 at the Phitsanulok site. Small blood samples (<0.5 mL) were collected from the brachial vein of each bird. A thin blood smear was then prepared, air-dried, and fixed in absolute methanol. Whole blood was transferred to 1.5 mL EDTA tubes and stored at −20 °C until further analysis. All samples were transported to The Monitoring and Surveillance Center for Zoonotic Diseases in Wildlife and Exotic Animals, Faculty of Veterinary Science, Mahidol University. All procedures involving wild pigeons were reviewed and approved by the Faculty of Veterinary Science, Animal Care, and Use Committee of Mahidol University (protocol no. MUVS-2016-03-09).

### 2.3 Staining and microscopic examination

Blood smears were stained using 1:20 diluted Giemsa's azur–eosin–methylene blue solution (Merck KGaA, Darmstadt, Germany) in phosphate buffer (pH 7.2) for 1 h according to the manufacturer's recommended protocol (Merck KGaA, 2021). Blood smears were then imaged under low magnification ( × 400) for 5–10 min and high magnification ( × 1000) for at least 100 fields using a Nikon/ECIPSE Ni–U Upright Microscope (Nikon Instruments Inc., New York, USA). The number of infected erythrocytes per 10,000 cells was used to estimate the intensity of infection (Godfrey et al., 1987). The morphometric characteristics of the infected erythrocytes were measured using NIS Elements imaging software (Nikon Instruments Inc., New York, USA). Identification of *Haemoproteus* parasites was based on a morphological characteristic key described by Valkiunas and Iezhova (2022).

### 2.4 DNA extraction and molecular analysis

DNA was extracted from the whole blood samples of wild pigeons. In total, 100 μL of purified DNA was eluted using a Genomic DNA Mini Kit (blood/cultured cells) (Geneaid Biotech, New Taipei City, Taiwan) according to the manufacturer's recommended protocol (Geneaid Biotech Ltd., 2002). The target nucleotide of the cytochrome *b* (*cyt b*) gene was amplified using nested polymerase chain reaction (PCR) with the HaemNF and HaemNR2 primers for the outer PCR product and the HaemF and HaemR2 primers for the inner nucleotide fragment, as previously described (Waldenstrom et al., 2004). For the outer DNA fragment, the PCR conditions were 30 s at 94 °C, followed by 20 cycles of amplification for 30 s at 94 °C, 30 s at 50 °C, 45 s at 72 °C, and a final extension of 10 min at 72 °C. The inner DNA fragments were amplified using 35 cycles under similar PCR conditions. In the nested step, PCR was performed using 1 μL of DNA template or PCR product, 1 unit of i-Taq™ DNA polymerase, 2 μL of 10 × PCR buffer and dNTP, 5 μM of each primer, and up to 20 μL of ultrapure water. Subsequently, 5 μL of amplified fragments were analyzed to determine the amplicon size by gel electrophoresis on 2% agarose gel using GelRed® (Biotium, CA, USA) and then visualized using a BioSens SC-Series 710 gel documentation system (GenXpress, Wiener Neudorf, Austria). DNA samples extracted from positive-microscopy slides and ultrapure water were used as positive and negative controls, respectively. Nucleotide sequencing of amplicons was performed by a commercial company (Bionics, Korea) using the Sanger sequencing method with both strands of inner primers.

### 2.5 DNA sequence analysis

The electropherograms of all sequences were carefully checked for wobble bases, which indicate co-infection, using BioEdit version 7.0.5.3 (Ibis Biosciences, CA, USA; Hall, 1999). Sequences were then subjected to a BLAST search using the NCBI GenBank and MalAvi databases (Bensch et al., 2009; NCBI, 2016).

### 2.6 Haplotype network and genetic analysis

Two different nucleotide alignments based on the 479 nucleotides of the partial *cyt b* gene of *H. columbae* were aligned and edited using MAFFT software (Katoh et al., 2019) and BioEdit version 7.0.5.3 (Hall, 1999). The first alignment was constructed using the 24 sequences newly obtained in this study, whereas the second alignment used the 267 sequences of *H. columbae* available in GenBank with a 99% identity similarity to the newly obtained sequences. The haplotype phases of partial *cyt b* sequences were inferred using PHASE (Stephens and Donnelly, 2003) within DnaSP version 6.12.03 (Rozas et al., 2017) and the following settings: 1000 iterations, 10 thinning interval, and 200 burn-in iterations using two alignments. Haplotype networks for *H. columbae* were constructed by the median-joining haplotype network using PopArt 1.7 (Bandelt et al., 1999; Leigh and Bryant, 2015). The first haplotype network constructed the relationship between *H. columbae* collected from the two study sites while the other reconstructed haplotype network related to the current worldwide distribution of *H. columbae* haplotypes. Genetic distances (*p*-distance) between the different haplotypes obtained were estimated using MEGA X (Kumar et al., 2018).

### 2.7 Data analysis

Comparisons of mean morphometric measurements between uninfected and infected erythrocytes, as well as between macro and microgametocyte, were performed using Student's independent *t*-test. A P value ≤ 0.05 was considered statistically significant.

## 3 Results

### 3.1 Detection and intensity of H. columbae infection

Haemosporidian infection was detected in 24 of 87 wild pigeons (*C. livia*) by both microscopic and molecular examinations. The positive infection rates at the Nakhon Sawan and Phitsanulok test sites were 22% (11/50) and 35% (13/37), respectively. The overall infection rate was approximately 27.6% (24/87). All positive samples underwent successful amplification of the partial *cyt b* gene. The obtained sequences were deposited in GenBank under accession numbers ON411230–ON411253. The intensity of haemosporidian infection was very low (approximately 0.028%; Table 1). The BLASTn results indicated 100% identity with one of the three common *H. columbae* lineages, namely HAECOL1, COLIV03, and COQUI05. HAECOL1 had a higher positive rate (18.4%) than COLIV03 and COQUI05, which had minor positive rates (4.6%).

**Table 1:** *Haemoproteus columbae* detected in 87 wild pigeons (*Columba livia*) in central Thailand and classified into three common haplotypes: HAECOL1, COLIV03, and COQUI05 (MalAvi lineages).

Province (Location); n. of sample	Sample ID	Parasitemia (%)	MalAvi lineage			Accession number (GenBank)
			HAECOL1	COLIV03	COQUI05	
Nakhon Sawan	NS001	0.011	0	0	1	ON411230
(15° 34′ N, 100° 13′ E);	NS008	0.001	0	1	0	ON411231
n. = 50	NS011	0.001	1	0	0	ON411232
	NS013	0.017	0	0	1	ON411233
	NS019	0.026	0	1	0	ON411234
	NS022	0.002	1	0	0	ON411235
	NS026	0.028	0	1	0	ON411236
	NS027	0.005	1	0	0	ON411237
	NS029	0.004	1	0	0	ON411238
	NS035	0.028	1	0	0	ON411239
	NS042	0.015	1	0	0	ON411240
Phitsanulok	PN003	0.093	1	0	0	ON411241
(16° 48′ N, 100° 15′ E);	PN004	0.002	0	1	0	ON411242
n = 37	PN006	0.038	1	0	0	ON411243
	PN012	0.004	1	0	0	ON411244
	PN013	0.079	1	0	0	ON411245
	PN014	0.168	1	0	0	ON411246
	PN016	0.016	0	0	1	ON411247
	PN021	0.019	1	0	0	ON411248
	PN022	0.028	0	0	1	ON411249
	PN032	0.015	1	0	0	ON411250
	PN033	0.030	1	0	0	ON411251
	PN035	0.026	1	0	0	ON411252
	PN037	0.032	1	0	0	ON411253
**Overall (n = 87)**						
n positive	24		16	4	4	
Positive rate (%)	27.6		18.4	4.6	4.6	

### 3.2 Morphological characteristics of H. columbae

**Fig. 1:**
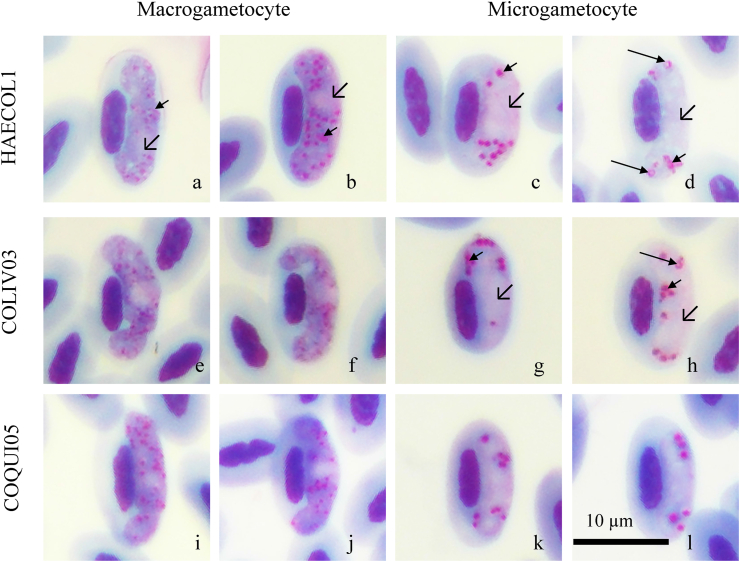
*Haemoproteus columbae* from the blood of wild pigeons (*Columba livia*); cytochrome *b* lineage HAECOL1 (a–d), COLIV03 (e–f) and COQUI05 (i–l); macrogametocytes (a–b; e–f; i–j) and microgametocytes (c–d; g–h; k–l); simple arrows: nuclei of erythrocytes; short triangle-head arrows: volutin granules; long triangle-head arrows: pigment granules; scale bar = 10 μm.

The gametocytes of *H. columbae* showed elongated growth along with the host nucleus and membrane of erythrocyte to both poles of erythrocytes, while fully grown gametocytes showed a predominantly halteridial form. The entire outline was found in mature gametocytes (Fig. 1a and b), but amoeboid form growth covered nearly both sites of the host nucleus pole found in immature gametocytes (Fig. 1e–f, i–j). They usually extended along the length and freely in the cytoplasm of erythrocytes and did not touch the host cell or nucleus membrane (Fig. 1a and b). The gametocyte nuclei were round to ovoid and stained pink, usually with the pigment and pose in the middle along with the host nucleus, resulting in the host nucleus being displaced to another side. Microgametocytes showed distinctly more displacement than macrogametocytes. Volutin granules were randomly distributed in the cytoplasm of fully grown gametocytes (Fig. 1a and b), with large discrete volutin granules possibly clumping into the group observed in microgametocytes (Fig. 1c–d, g–h). A smaller pigment area was usually found in microgametocytes. Pigment granules (hemozoin) were observed inside the volutin granules of fully grown microgametocyte (Fig. 1d and h). The gametocytes showed dimorphism, with the cytoplasm having a dark blue color and distinctly small pink nucleus of macrogametocytes, but pale staining with large pale pink nuclei in microgametocytes (Fig. 1).

The morphometry of uninfected and infected erythrocyte with macro- and microgametocyte are shown in Table 2. Erythrocyte infection with *H. columbae* resulted in significant hypertrophy in all three dimensions (all P < 0.01): length, width, and area of infected erythrocytes. In contrast, the nuclei of erythrocytes showed less impact from *H. columbae* infection, but the atrophied width of the nuclei may have indicated macrogametocyte infection (P < 0.05). Erythrocyte distortion of each infection of the three *cyt b* lineages was consistent with the effect of *H. columbae*, although COQUI05 infection showed minor variation with a significantly different length and area of the host nucleus (shown in Supplementary Fig. 1). Comparisons between macro- and microgametocytes, the length of erythrocytes, and their nuclei revealed different impacts of infection. Furthermore, dimorphism was observed among the dimension of gametocytes in terms of their nucleus and number of pigments, but not the width of gametocytes (P = 0.54), as shown in Table 2.

**Table 2:** Morphometry data of *Haemoproteus columbae* from wild pigeons (*Columba livia*) in Thailand. Data are reported as the mean ± standard deviation (SD) and range (in μm).

Measurement	Uninfected erythrocytes (n = 44)		Macrogametocytes (n = 59)		Microgametocytes (n = 42)	
	Mean ± SD	Range	Mean ± SD	Range	Mean ± SD	Range
**Erythrocyte**						
Length	9.2 ± 0.6	8.6–9.7	10.0 ± 0.6	9.4–10.6	9.7 ± 0.6	9.2–10.3
Width	5.2 ± 0.3	4.9–5.5	5.6 ± 0.4	5.2–6.0	5.6 ± 0.4	5.2–6.0
Area	37.3 ± 2.6	34.7–39.9	43.5 ± 2.9	40.6–46.4	42.3 ± 3.2	39.1–45.4
**Erythrocyte nucleus**						
Length	4.6 ± 0.3	4.3–4.9	4.5 ± 0.3	4.1–4.8	4.6 ± 0.4	4.2–5.0
Width	1.9 ± 0.2	1.7–2.1	1.8 ± 0.1	1.6–1.9	1.8 ± 0.1	1.7–2.0
Area	6.6 ± 0.6	6.0–7.2	6.6 ± 0.8	5.8–7.3	6.8 ± 0.9	5.9–7.6
NDR^a^	–	–	0.60 ± 0.06	0.53–0.65	0.60 ± 0.06	0.53–0.65
**Gametocyte**						
Length	–	–	9.2 ± 0.6	8.6–9.9	8.7 ± 0.7	8.0–9.4
Width	–	–	2.7 ± 0.3	2.4–3.0	2.7 ± 0.3	2.5–3.0
Area	–	–	23.0 ± 2.6	20.4–25.6	20.7 ± 2.7	18.1–23.4
Pigment granule	–	–	29.8 ± 5.4	24.5–35.2	10.1 ± 4.0	6.1–14.1
**Gametocyte nucleus**						
Length	–	–	2.2 ± 0.6	1.6–2.8	4.7 ± 1.0	3.7–5.7
Width	–	–	2.0 ± 0.4	1.6–2.4	2.3 ± 0.3	2.0–2.6
Area	–	–	3.8 ± 0.8	3.0–4.6	8.7 ± 2.3	6.4–11.1

a NDR: nucleus displacement ratio was calculated by 2X/(X + Y), where X and Y are the distance from the periphery of host cell to that of the host nucleus without and with gametocytes, respectively, as described by Bennett and Campbell (1972).

### 3.3 Molecular characteristics and haplotype network of H. columbae

**Fig. 2:**
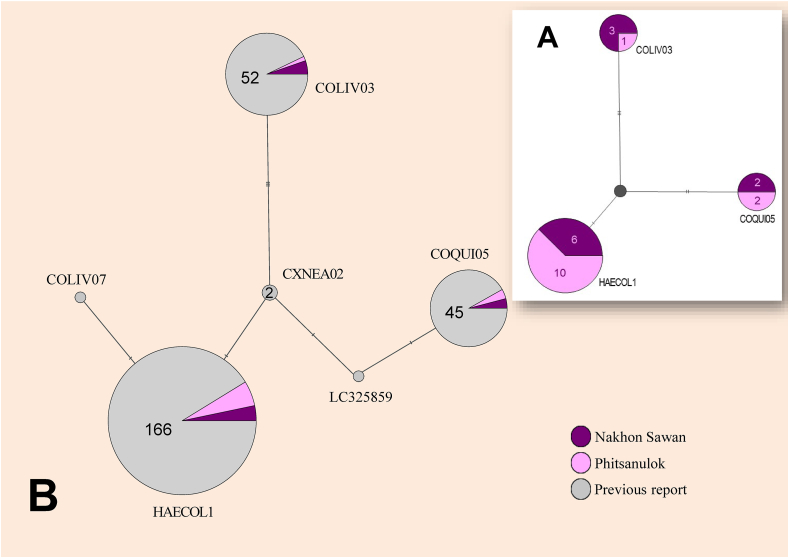
Haplotype network of partial *cyt b* sequence (479 bp) of *Haemoproteus columbae* from wild pigeons (*Columba livia*); (A) the three common haplotypes (HAECOL1, COLIV03 and COQUI05) found in this study and proportions between study sites; (B) proportions of each haplotype reported worldwide and in the two study sites; Nakhon Sawan (purple), Phitsanulok (pink), and previous reports (gray); number of sequences shown in a pie chart of the network, without number inside indicates one sequence. (For interpretation of the references to color in this figure legend, the reader is referred to the Web version of this article.)

Networks of partial *cyt b* gene sequences (Fig. 2A) revealed that *H columbae* could be divided into three haplotypes. HAECOL1 had the highest frequency (16 sequences) and was a connected node with a single nucleotide. This node constructed a tri-way linked formation; a second one was linked directly to COQUI05 with double nucleotide variation and last one build to COLIV03 with three nucleotides. The frequency of the two minor haplotypes was not different (n = 4 sequences each). A network of previously reported haplotypes and the current network are shown in Fig. 2B. This network contained six haplotypes. HAECOL1 was a major haplotype with the highest frequency that form two single nucleotide variations: one branch with COLIV07 and another branch with CXNEA02. However, CXNEA02 acted as a middle point of nucleotide variation among HAECOL1, COLIV03, and COQUI05. One branch of CXNEA02 linked to COLIV03 with three nucleotides and another branch linked to an unnamed haplotype (GenBank no.: LC325859). This unnamed haplotype link serves as a connected point between CXNEA02 and COQUI05 with individual single variable nucleotides. Both networks (Fig. 2A and B) showed that *H. columbae* can be classified into at least three common haplotypes, as well as those found in this study. Furthermore, this 479 bp. partial *cyt b* gene of *H. columbae* with other available in GenBank; showed 6 variable sites at the following nucleotide positions: 153, 186, 258, 306, 312 and 387. Among the three common haplotypes, COQUI05 and COLIV03 represented three and four variable sites based on HAECOL1 nucleotide variation, whereas the other haplotype had a one and two nucleotide difference, as shown in Table 3. In the evolution divergence (*p*-distance) analysis among common haplotypes of *H. columbae* based on partial 479 bp *cyt b* gene sequence, the highest distance was observed between COLIV03 and COQUI05 (approximately 1%) and the lowest was found between HAECOL1 and COLIV03 (0.8%) and between HAECOL1 and COQUI05 (0.6%) (Table 4).

**Table 3:** Differences in the partial *cyt b* gene (479 bp) sequence based on reference lineages in the MalAvi database from *Haemoproteus columbae* haplotypes reported here and worldwide.

Haplotype	Point mutation					
	nt_153	nt_186	nt_258	nt_306	nt_312	nt_387
HAECOL1	T	G	T	A	C	A
COQUI05	.	.	.	T	T	G
COLIV03	A	T	A	.	.	G
COLIV07	.	A	.	.	.	.
CXNEA02	.	.	.	.	.	G
Unnamed^a^	.	.	.	T	.	G

a Not available in MalAvi database (GenBank accession code LC325859).

**Table 4:** Estimates of evolutionary divergence (*p*-distance; %) between partial *cyt b* (479 bp) gene sequences of common haplotypes of *Haemoproteus columbae*.

	HAECOL1	COLIV03	COQUI05
**HAECOL1**			
**COLIV03**	0.835		
**COQUI05**	0.626	1.044	

## 4 Discussion

Haemosporidian parasites are one of the causes of mortality and fatality in some bird species. However, there are limited reports of haemosporidian infection of bird species in Thailand, with only one publication reporting eight *Haemoproteus* morphospecies in a wetland of Bung Boraphet, Nakhon Sawan province (Prompiram et al., 2015). In the present study, a common species—namely the wild pigeon (*Columba livia*)— was studied to enable haemosporidian parasite identification. Microscopic and molecular methods were applied to characterize the haemosporidian parasite. This information regarding haemosporidian parasites can serve as useful primary data for taxonomy and diversity research in this region.

In this study, wild pigeons had a 27.6% infection rate with haemosporidian parasite (Table 1), which was similar to the relatively low prevalence rates reported for domestic pigeons in Iran (Adinehbeigi et al., 2018; Dehghani Samani et al., 2013) and wild pigeons in Japan (Yumoto et al., 2021). However, the rate of haemosporidian parasite infection can be higher — especially in urban areas or pigeon farms — such as the >95% prevalence in urban areas of South Africa (Nebel et al., 2020) and 65%–100% in pigeon farms in Indonesia (Rosyadi et al., 2021). This difference infection rate is likely associated with population density and habitat area. A high infection rate is generally associated with a high density and restricted foraging areas due to urbanization. This setting encourages disease infection and transmission, including that of vector-borne parasites (Woodworth et al., 2005). The bird density at the lowland site in the present study has been estimated to range from 123.4 to 961.3 birds/km^2^ (Chamchumroon et al., 2013). It has also been established that the density and number of pigeon flocks in this region is related to the abundance of buildings, with a low density in agricultural fields (434 birds/km^2^) and a high density in urban areas (up to 2083 birds/km^2^) (Sacchi et al., 2002). The open area of paddy fields could provide an individual foraging area or at least separate the birds into small groups, consequently reducing parasite transmission.

Based on morphological characteristics and morphometry (Fig. 1 and Table 2), the haemosporidian parasite was identified as *Haemoproteus columbae*. Infection of erythrocyte with *H. columbae* was distorted on all dimension (P < 0.01); length, width, and area, but showing less affected the nucleus of erythrocyte. This morphological character is similar to a previous description of *H. columbae* based on pooled measurements from six locations on different continents (Bennett and Peirce, 1990). Conversely, gametocytes showed a halteridial form, entire outline with amoeboid in young gametocytes, which markedly displaced the erythrocyte nucleus (nucleus displacement ratio (NDR) = 0.5–0.6), consistent with previous reports of morphologic characters (Krizanauskiene et al., 2013). Moreover, distinct dimorphism between gametocytes using staining or morphological measurement is possible. In addition, the proportion of pigment between macrogametocytes (an average of 30) and microgametocytes was nearly two-fold. The number of pigments was different to *H. multipigmentatus* which showed more pigments, i.e., approximately 40 (Valkiunas et al., 2010).

A high frequency of infection with HAECOL1 relative to other haplotypes was observed in this study, which is similar to previous findings in wild or domestic pigeons (Cepeda et al., 2019; Chagas et al., 2016; Nebel et al., 2020; Rosyadi et al., 2021; Yumoto et al., 2021). Notably, COLIV03 and COQUI05 had minor infection rates (Nebel et al., 2020; Rosyadi et al., 2021), consistent with those reported previously. The presence of all three haplotypes has been reported around the world, including the current finding in Thailand (Table 1). Therefore, these three could be considered the common haplotypes of *H. columbae*. The haplotype network represents the genetic relationships of *H. columbae* in current or previous reports. HAECOL1 has been recorded in different geographic areas, including Botswana, Brazil, Colombia, Indonesia, Iran, Italy, Japan, and South Africa (Cepeda et al., 2019; Chagas et al., 2016; Dehghani Samani et al., 2013; Mushi et al., 2000; Rosyadi et al., 2021; Yumoto et al., 2021). This wide distribution of HAECOL1 is similar to that of COLIV03 and COQUI05. The rare haplotype of *H. columbae* possibly showed a limited distribution, similar to COLIV07 and an unnamed haplotype (GenBank no.: LC325859) reported in pigeons from Peru (Pacheco et al., 2018) and Japan. Of the three haplotypes of *H. columbae* obtained, analysis revealed a 3–4 nucleotide variation and 0.6%–1.0% genetic distance. Genetic differentiation of *H. columbae* cannot distinguish morphological characters among the different haplotypes. Although previous studies hypothesized that >5% genetic dissimilarity in the *cytb* gene is indicative with different morphospecies (Hellgren et al., 2007), later studies have shown that this might not be a reliable criterion to differentiate morphospecies of avian haemosporidians (de Freitas et al., 2023; Palinauskas et al., 2015). This genetic difference was similar to *H. columbae* reported in urban pigeons in South Africa (Nebel et al., 2020). However, *H. columbae* was not the sole haemosporidian parasite; other *Haemoproteus* spp. were also found in pigeons, such as COLIV06. This haplotype showed 10%–11% genetic distance from *H. columbae* reported in Sao Paulo Zoo, Brazil and Cape Town, South Africa (Chagas et al., 2016; Nebel et al., 2020); unfortunately, no other *Haemoproteus* parasite was found in the present study. Overall, this study showed that the haplotype diversity of *H. columbae* in wild pigeons from lowland areas of a tropical region was similar to that reported in other studies worldwide. These results suggest that geographical and environmental factors have little influence on the genetics of *H. columbae*. Thus, this study reports the characteristic morphology and genetics of *H. columbae* in the specific ecological context of low land paddy fields in Thailand.

## 5 Conclusions

This study provides the first report of morphological and molecular characteristics of the haemosporidian parasite in wild pigeons (*C. livia*) in Thailand. This parasite was characterized by common species such as *H. columbae* with three common lineages, namely HAECOL1, COLIV03, and COQUI05. This work provides essential regional knowledge about haemosporidian parasites for future phylogeographical research. Knowledge of wildlife diseases in this tropical region should be expanded to explore pathogenic infections in new ecological and geographic distributions in endemic host species, which could provide essential information for disease prevention or species conservation.

## Authors’ contribution

Conceptualization, P.P., K.P., T.C.; methodology, P.P., K.P.; formal analysis, P.P., K.P.; investigation, P.P., C.M., K.P., S.C., N.S.; writing—original draft preparation, P.P., T.C.; All authors have read and agreed to the published version of the manuscript.

## Funding

This research project was supported by 10.13039/501100004704National Research Council of Thailand (NRCT) and 10.13039/501100004156Mahidol University (Sub-Code: NRCT5-TRG63009-08).

## Ethical approval/animal welfare statement

The study was reviewed and approved by the Faculty of Veterinary Science – Animal Care and Use Committee (FVS-ACUC) of Mahidol University (protocol no. MUVS-2016-03-09).

## Declaration of competing interest

The authors declare no conflict of interest.
