# Supplementary material for: Baseline study of the morphological and genetic characteristics of Haemoproteus parasites in wild pigeons (Columba livia) from paddy fields in Thailand
Source: Int J Parasitol Parasites Wildl. 2023 Apr 11;21:153–9. doi: 10.1016/j.ijppaw.2023.04.003 (PMC10205435; doi:10.1016/j.ijppaw.2023.04.003)
Supplement: Multimedia component 1 [file mmc1.pdf]

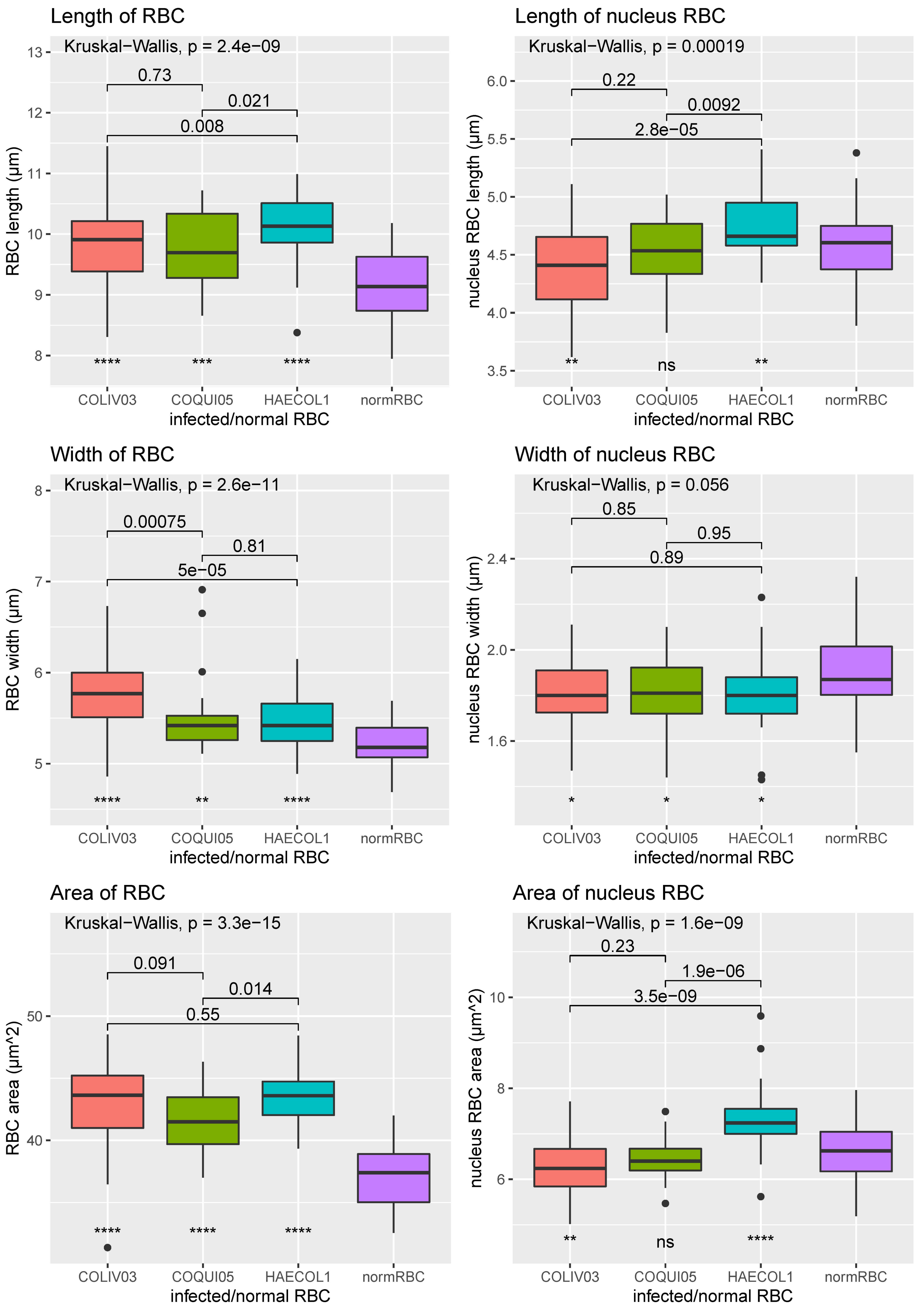

Length of RBC
Kruskal−Wallis, p = 2.4e−09
0.73
0.021
0.008
****
***
****
RBC length (µm)
COLIV03
COQUI05
HAECOL1
normRBC
infected/normal RBC
Length of nucleus RBC
Kruskal−Wallis, p = 0.00019
0.22
0.0092
2.8e−05
**
ns
**
nucleus RBC length (µm)
Width of RBC
Kruskal−Wallis, p = 2.6e−11
0.00075
0.81
5e−05
****
**
****
RBC width (µm)
Width of nucleus RBC
Kruskal−Wallis, p = 0.056
0.85
0.95
0.89
*
*
*
nucleus RBC width (µm)
Area of RBC
Kruskal−Wallis, p = 3.3e−15
0.091
0.014
0.55
****
****
****
RBC area (µm^2)
Area of nucleus RBC
Kruskal−Wallis, p = 1.6e−09
0.23
1.9e−06
3.5e−09
**
ns
****
nucleus RBC area (µm^2)
